# Buoyancy forcing and the subpolar Atlantic meridional overturning circulation

**DOI:** 10.1098/rsta.2022.0181

**Published:** 2023-12-11

**Authors:** Martha W. Buckley, M. Susan Lozier, Damien Desbruyères, Dafydd Gwyn Evans

**Affiliations:** ^1^ Department of Atmospheric, Oceanic and Earth Sciences,George Mason University, Fairfax, VA 22030, USA; ^2^ School of Earth and Atmospheric Sciences, Georgia Institute of Technology, Atlanta, GA 30318, USA; ^3^ Laboratoire d’Océanographie Physique et Spatiale (LOPS),Univ Brest, CNRS, Ifremer, IRD, IUEM, F29280 Plouzané, France; ^4^ National Oceanography Centre, European Way, Southampton, SO14 3ZH, UK

**Keywords:** Atlantic meridional overturning circulation, buoyancy forcing, water mass transformation

## Abstract

The North Atlantic meridional overturning circulation and its variability are examined in terms of the overturning in density space and diapycnal water mass transformation. The magnitude of the mean overturning is similar to the surface water mass transformation, but the density and properties of these waters are modified by diapycnal mixing. Surface waters are progressively densified while circulating cyclonically around the subpolar gyre, with the densest waters and deepest convection occurring in the Labrador Sea and Nordic Seas. The eddy-driven interaction between the convective interior and boundary currents is a key to the export of dense waters from marginal seas. Due to the multitude of pathways of dense waters within the subpolar gyre, as well as mixing with older waters, waters exiting the subpolar gyre have a wide range of ages, with a mean age on the order of a decade. As a result, interannual changes in water mass transformation are mostly balanced locally and do not result in changes in export to the subtropics. Only persistent changes in water mass transformation result in changes in export to the subtropics. The dilution of signals from upstream water mass transformation suggests that variability in export of dense waters to the subtropics may be controlled by other processes, including interaction of dense waters with the energetic upper ocean.

This article is part of a discussion meeting issue ‘Atlantic overturning: new observations and challenges’.

## 1. Introduction

There are only a few places in the world ocean where wintertime cooling is able to erode the stratification and produce deep-reaching convection. Deep convection occurs in the North Atlantic, rather than the North Pacific, because surface waters in the Atlantic are saltier than the Pacific [1–3]. Dense waters formed in the North Atlantic, as well as adjacent marginal seas, are generally referred to as North Atlantic Deep Water (NADW). Deep convection and dense water formation also occur around Antarctica, and waters formed there fill the abyssal ocean. NADW flows southward at depth in both the deep western boundary current (DWBC) [4,5] and interior pathways [6–9]. This southward flow at depth is compensated by the northward surface-intensified flow.

**Figure 1.**
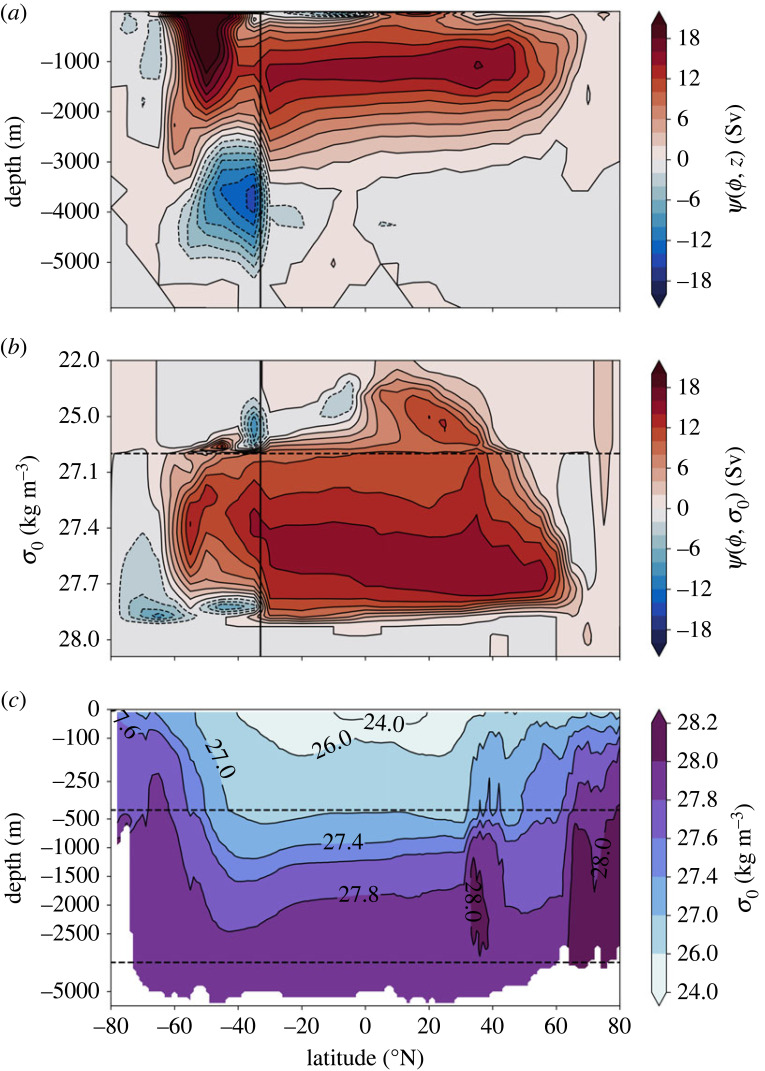
(*a*,*b*) The mean Atlantic overturning streamfunction in (*a*) depth coordinates, Ψ(ϕ,z), and (*b*) density coordinates, Ψ(ϕ,σ), calculated using the Eulerian meridional velocity plus the bolus velocity from ECCO v4 [10] over the Atlantic sector north of $33^{\circ }\;S$ (marked by the black vertical line) and over the full Southern Ocean south of $33^{\circ }\;S$. A comparison of overturning at the OSNAP array between ECCO v4 and OSNAP observations is shown in electronic supplementary material, figure S1. (*c*) The mean potential density zonally averaged over the Atlantic sector, from ECCO v4 [10]. In all plots, potential density is referenced to the surface ($\sigma_{0}$). The horizontal dashed lines in (*b*) and (*c*) mark a change in the scale of the y-axis tick intervals.

As a result of this surface to mid-depth overturning (figure 1*a*,*b*), generally referred to as the Atlantic Meriodional Overturning Circulation (AMOC), the Atlantic transports heat northward in both hemispheres, with a 0.5 PW cross-equatorial ocean heat transport (OHT) (see Figure 3*b* in [11] and Fig. 1 in [12]). OHT also extends much further north in the Atlantic than in the Pacific, and the Atlantic is warmer than the Pacific at the same latitudes (see Fig. 3*c* in [13]). Numerous observational programs are dedicated to observing the AMOC and the associated heat and freshwater transports, including the RAPID mooring array deployed at $26.5^{\circ }\;\text{N}$ in 2004 [14,15] and the OSNAP mooring array deployed in the subpolar North Atlantic in 2014 [16].

In order for an adiabatic overturning to be sustained, there must be shared isopycnal outcrops between the northern hemisphere high latitudes and the Southern Ocean [17], where dense waters upwell adiabatically to the surface due to the prevailing westerly wind stress [18,19]. This condition is met in the North Atlantic (figure 1*c*) but not in the North Pacific (see Figure 1 in [17]), and, as a result, the surface to mid-depth overturning is restricted to the Atlantic basin.

The AMOC is typically quantified as an overturning streamfunction in latitude-depth or latitude-density coordinates (see figure 1*a*,*b* and §2). Many of the historical theoretical [20–24] and modelling [25–27] studies focus on the overturning in latitude-depth coordinates. However, because ocean currents tend to follow isopycnal surfaces, the overturning in density coordinates provides a more accurate description of the flow field than the overturning in depth space (see §2a and figure 1*b*). An added benefit of consideration of the overturning in density space is that this overturning can be connected, via volume and buoyancy budgets, to diapycnal water mass transformation using the Walin framework [28] (see §2b).

The purpose of this article is to reexamine the structure of the mean AMOC and its variability in terms of the overturning in density space and the role of diapycnal water mass transformation. It should be noted, in advance, that the focus on the overturning in density coordinates implies a strong relationship between the mean diapycnal water mass transformation and the mean overturning (§2b). Yet, this is still consistent with the Southern Ocean winds providing the energy needed to sustain the large-scale overturning. Both local and remote processes are important for surface water mass transformation in the subpolar North Atlantic, as in the mean, buoyancy loss over the subpolar North Atlantic must be balanced by buoyancy gain elsewhere, including the warming of waters upwelled in the Southern Ocean.

In §2a, we describe the overturning in depth and density coordinates, highlighting the differences between the two and showing that a density coordinate framework is preferable. Furthermore, in §2b, we highlight the intimate connection between the overturning in density space and diapycnal water mass transformation. In §3a, we contrast the geography of surface water mass transformation (densification of surface waters) with the geography of deep-reaching convection. In addition, we show that the eddy-driven interaction between the convective interior and the boundary current is essential for water mass transformation, subduction and the overturning in density space (§3b). In §4, we reexamine the relationship between variations in upstream water mass production and downstream export in the context of the Walin framework. We find that most of the surface water mass transformation on intraannual to interannual timescales is balanced by changes in volume between isopycnals rather than export, which explains the weak relationships between water mass production and overturning found previously [7,8]. In §5, we conclude and offer suggestions for future work.

## 2. Overturning diagnostics

In this section, we describe diagnostics used to quantify the AMOC, and the challenges inherent in attempting to collapse a complex, three-dimensional circulation into two dimensions.

### (a) Defining the Atlantic meridional overturning circulation

The overturning circulation can be described mathematically as a zonally integrated overturning streamfunction. The zonally integrated meridional volume transport in depth coordinates is given by

2.1 $$\Psi (\phi ,z,t)=-\int_{z_{b}}^{z}\int_{x_{w}}^{x_{e}}v\;dx\;dz,$$

where v is the meridional velocity (or the velocity perpendicular to a given section), z is a vertical coordinate increasing upwards, $z_{b}$ is the depth of the bottom and $x_{w}(\phi ,z)$ and $x_{e}(\phi ,z)$ are the westward and eastward positions of the bathymetry at a particular latitude (ϕ) and depth. The time mean Ψ(ϕ,z) in the Estimating the Circulation and Climate of the Ocean (ECCO [10]) estimate shows a northward near-surface flow and a southward flow between about 1000 and 3000 m depth (figure 1*a*).

In the subtropical Atlantic, most of the OHT results from the overturning circulation in the latitude-depth plane, as the surface to deep temperature contrasts are much larger than the east-west temperature contrast of the gyre circulation. However, in the subpolar North Atlantic isopycnals slope upward from east to west, and a significant portion of the OHT results from the flow of warm, near-surface waters northward in the eastern basin and cold waters southward in the western basin at the same depth [29]. At the OSNAP array in the subpolar North Atlantic (see red line in figure 3*a*), nearly all the OHT is due to a horizontal circulation rather than an overturning in depth space [16]. In addition, as ocean currents flow along isopycnal surfaces, zonal averages along isopycnals surfaces are a more accurate characterization of the flow field than averages in depth coordinates. For these reasons, the overturning streamfunction in density coordinates is now favoured, at least for quantifying the AMOC and its associated transport of heat or buoyancy in the subpolar gyre. The overturning streamfunction in density coordinates is defined as follows:

2.2 $$\Psi (\phi ,\sigma ,t)=-\int_{\sigma_{b}}^{\sigma }\int_{x_{w}}^{x_{e}}v\;dx\;d\sigma ,$$

where $\sigma_{b}$ is the maximum (bottom) density at latitude ϕ and $x_{w}(\phi ,\sigma )$ and $x_{e}(\phi ,\sigma )$ are the westward and eastward positions, respectively, of the bathymetry at a particular latitude and density. The time mean overturning streamfunction in the subpolar North Atlantic (north of $40^{\circ }\;\text{N}$) is substantially stronger in density coordinates than in depth coordinates (figure 1*b*), as it captures warm northward flowing waters which are cooled substantially before returning southward at the same depth. At the OSNAP array, 73% of the mean Atlantic OHT and 87% of monthly variance of Atlantic OHT are captured by the overturning in density space [16].

The strength of the overturning in density space is defined as follows:

2.3 $$\Psi_{\max}(\phi ,t)=\max[\Psi (\phi ,\sigma ,t)]=-\int_{\sigma_{b}}^{\sigma_{\mathrm{MOC}}}\int_{x_{w}}^{x_{e}}v\;dx\;d\sigma ,$$

where $\sigma_{\mathrm{MOC}}$ is the density at which Ψ(ϕ,σ,t) is maximum. This quantity is sometimes referred to as the volume transport of the lower limb, although at some subpolar latitudes these waters may be contained within the mixed layer.

Overturning streamfunctions can also be calculated in temperature and salinity space, defined analogously to equation (2.2), but with σ replaced with potential temperature, θ, or salinity, S. These streamfuctions are relevant to the ocean heat and freshwater transports, respectively [30], and they may differ substantially from Ψ(ϕ,σ). For instance, the overturning in the Labrador Sea is weak in density space, but much larger in temperature and salinity space. The cooling and freshening of surface waters as they transit the Labrador Sea largely compensate and thus have a small impact on density [31,32]. In the Labrador Sea (across OSNAP West), the overturning and isopycnal circulation contribute equally to the mean and monthly variability of Atlantic OHT [33].

In summary, zonally integrated overturning streamfunctions provide a mathematically convenient definition of the AMOC. Yet, there are substantial challenges inherent in attempting to collapse a complex, three-dimensional circulation into two-dimensional circulation. The overturning streamfunction depends on the vertical coordinate utilized (depth, density, temperature, and salinity), particularly in the subpolar North Atlantic.

### (b) Water mass transformation and the overturning in density space

An added advantage of considering the overturning in density space is that Ψ(ϕ,σ) is tied to diapycnal water mass transformation through volume and buoyancy budgets, often called the Walin framework [28,34–37]. The Walin framework is ideal for an investigation of the processes which set the mean strength of the overturning (discussed in this section) and of understanding variations in the overturning (§4).

The volume and buoyancy budget of a layer below isopycnal σ enclosed within a geographical domain between $\phi_{1}$ and $\phi_{2}$ implies

2.4 $$F(\phi_{1}<\phi <\phi_{2},\sigma )+G_{D}(\phi_{1}<\phi <\phi_{2},\sigma )=\frac{\partial V(\phi_{1}<\phi <\phi_{2},\sigma )}{\partial t}+\Psi (\phi_{1},\sigma )-\Psi (\phi_{2},\sigma ),$$

where $V(\phi_{1}<\phi <\phi_{2},\sigma )$ is the volume of fluid denser than σ between latitudes $\phi_{1}$ and $\phi_{2}$. $F(\phi_{1}<\phi <\phi_{2},\sigma )$ and $G_{D}(\phi_{1}<\phi <\phi_{2},\sigma )$ are the diapycnal volume fluxes crossing the isopycnal σ between latitudes $\phi_{1}$ and $\phi_{2}$ due to air–sea buoyancy fluxes and mixing, respectively. The sign convention is that F and $G_{D}$ are positively directed towards increasing σ, and, in order to be precise, we explicitly include the latitude range over which F and $G_{D}$ are evaluated. $G_{D}$ includes all mixing processes, including those occurring within the mixed layer, such as mixed layer entrainment [38,39]. In accord with equation (2.4), diapycnal water mass transformation can lead to volume inflation and/or export across $\phi_{1}$ and $\phi_{2}$.

In discrete form F, generally referred to as the surface water mass transformation, is given by

2.5 $$F(\phi_{2}<\phi <\phi_{2},\sigma )=\frac{1}{\Delta \sigma }\iint_{\phi_{1}<\phi <\phi_{2}}F(x,y,\sigma )\;dx\;dy.$$

F(x,y,σ) is a two-dimensional map of the transformation across σ [40]:

2.6 $$F(x,y,\sigma )=[\frac{\alpha }{C_{p}}Q+\beta \frac{S}{1-S}(E-P)]\Pi (\sigma^{\prime }(x,y)),$$

where α is the thermal expansion coefficient, β is the haline contraction coefficient, S is the surface salinity, $C_{p}$ is the specific heat, Q is the heat flux into the ocean, and E and P are the evaporation and precipitation, respectively. $\Pi (\sigma^{\prime }(x,y))$ selects the outcrop of density σ and is given by

$$\Pi (\sigma^{\prime }(x,y))=\left\{ \begin{array}{ll} 1, & |\sigma^{\prime }(x,y)-\sigma |\leq \frac{\Delta \sigma }{2}\\ 0, & \text{elsewhere}. \end{array}\right.$$

Here, we quantify the relationship between surface water mass transformation, water mass transformation due to mixing, and overturning between $45^{\circ }\;\text{N}$ and the Greenland–Scotland Ridge/Davis Strait (northern boundary denoated as $\phi_{N}$) using Estimating the Circulation and Climate of the Ocean version 4 (ECCO v4, [10]). ECCOv4 is well suited for understanding volume and density budgets because it preserves properties exactly (unlike sequential reanalyses), enabling the mathematical quantification of all terms in the Walin framework. Since $G_{D}$ is often calculated as a residual, it will include errors unless applied to a model with closed volume and buoyancy budgets, such as ECCO v4 [41]. Due to its coarse resolution (nominally $1^{\circ }$), ECCO v4 may not accurately capture the interior-boundary eddy fluxes important for the overturning in convective basins and suffers from biases in the subpolar North Atlantic typical of coarse-resolution models.

**Figure 2.**
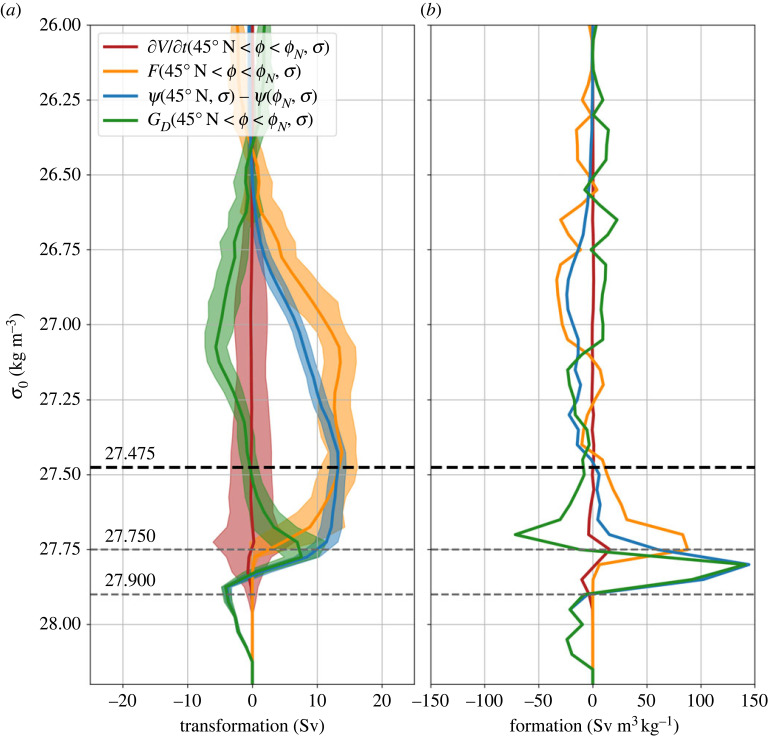
(*a*) Time-mean diapycnal water mass transformation and overturning for the region between $45^{\circ }\;\text{N}$ and the Greenland–Scotland Ridge/Davis Strait ($\phi_{N}$) from Estimating the Circulation and Climate of the Ocean version 4 (ECCO v4) [10]. The plot shows the surface water mass transformation, $F(45^{\circ }\;\text{N}<\phi <\phi_{N},\sigma )$ (orange line); the overturning, $\Psi (45^{\circ }\;\text{N},\sigma )-\Psi (\phi_{N},\sigma )$ (blue line); and the volume change below isopycnal σ, $\partial V/\partial t(45^{\circ }\;\text{N}<\phi <\phi_{N},\sigma )$ (red line). The green line indicates the residual, which is interpreted as the diapycnal water mass transformation due to mixing, $G_{D}(45^{\circ }\;\text{N}<\phi <\phi_{N},\sigma )$. The shading represents the standard deviation of the annual means. The dashed black line is at the density of the maximum overturning, $\sigma_{\mathrm{MOC}}$. The overturning is shown separately for $45^{\circ }\;\text{N}$ and the Greenland–Scotland Ridge in electronic supplementary material, figure S1. (*b*) The convergence in density space of the terms in (*a*), with positive values indicating that a given term creates waters within a density class.

Between $45^{\circ }\;\text{N}$ and $\phi_{N}$, the general structure of the overturning, $\Psi (45^{\circ }\;\text{N},\sigma )-\Psi (\phi_{N},\sigma )$, is similar to that of the surface water mass transformation, $F(45^{\circ }\;\text{N}<\phi <\phi_{N},\sigma$) (figure 2*a*). The strength of the overturning ($\Psi_{\max}$, equation (2.3)) is nearly identical to the strength of the surface water mass transformation across $\sigma_{\mathrm{MOC}}$. However, the peak overturning is at a higher density than that of the surface water mass formation (figure 2*a*), as noted previously by [41–43]. Using an inverse box model constrained by observations, [44] previously showed the importance of diapycnal mixing in water mass transformation between $48^{\circ }\;\text{N}$ and the Greenland–Scotland Ridge. Using Lagrangian pathways computed in a high-resolution ocean mode, [45] showed the importance of diapycnal mixing for NADW exported in the boundary current at $53^{\circ }\;\text{N}$. Our results for ECCO v4 are consistent with observational and modelling results showing that at OSNAP the total volume transport of waters in the lower limb has a magnitude similar to that of the surface water mass transformation, while diaypcnal mixing leads to modest modifications of the density structure and large changes in the temperature/salinity structure of the lower limb [41,43,46].

The role of surface fluxes and mixing in creating/destroying water masses within an infintesimal layer dσ, as well as the transport in this layer per unit density, may be seen from the convergence in density space of the terms in the water mass transformation equation (taking −∂/∂σ of equation (2.4)). Between $45^{\circ }\;\text{N}$ and $\phi_{N}$, air–sea fluxes produce dense lower limb waters, with densities up to $\sigma_{0}=27.850{\text{ kg m}}^{-3}$. Mixing destroys light NADW and dense overflow waters (OWs), mixing these waters to produce NADW with intermediate densities (figure 2*b*).

The equation describing water mass formation in a discrete layer between two isopycnals $\sigma_{1}$ and $\sigma_{2}$ (over the domain $\phi_{1}<\phi <\phi_{2}$) can be written as follows:

2.7 $$\Delta F+\Delta G_{D}=\frac{\partial \Delta V}{\partial t}+\Delta \Psi (\phi_{1})-\Delta \Psi (\phi_{2}),$$

where $\Delta F=F(\phi_{1}<\phi <\phi_{2},\sigma_{1})-F(\phi_{1}<\phi <\phi_{2},\sigma_{2})$ is the surface water mass formation and $\Delta G_{D}(\phi_{1}<\phi <\phi_{2},\sigma_{1})-G_{D}(\phi_{1}<\phi <\phi_{2},\sigma_{2})$ is the water mass formation due to mixing. ΔV is the volume of the layer $\sigma_{1}<\sigma <\sigma_{2}$ between $\phi_{1}$ and $\phi_{2}$. $\Delta \Psi (\phi )=\Psi (\phi ,\sigma_{1})-\Psi (\phi ,\sigma_{2})$ is the volume transport in the layer $\sigma_{1}<\sigma <\sigma_{2}$; thus, $\Delta \Psi (\phi_{1})-\Delta (\Psi \phi_{2})$ is the volume transport convergence in this layer.

We now consider the water mass formation in specific $\sigma_{0}$ layers between $45^{\circ }\;\text{N}$ and $\phi_{N}$ using ECCO v4. Despite large production of light NADW ($27.475<\sigma_{0}<27.750{\text{ kg m}}^{-3}$) by air–sea fluxes (ΔF=10.9 Sv), most of these waters are destroyed by mixing ($\Delta G_{D}=-7.6\;\mathrm{Sv}$), and the transport convergence in this density layer is only $\Delta \Psi (45^{\circ }\;\text{N})-\Delta \Psi (\phi_{N})=3.3\;\mathrm{Sv}$. Mixing also destroys about 3.9 Sv of the densest OWs ($\sigma_{0}>27.900{\text{ kg m}}^{-3}$). Mixing of light NADW and dense OWs leads to the production of 11.1 Sv of waters with densities $27.750<\sigma_{0}<27.900{\text{ kg m}}^{-3}$, which combined with a small contribution due to air–sea fluxes (ΔF=2.5 Sv), leads to a 13.7 Sv transport convergence in this density range. It is this production of dense NADW by mixing that results in a peak overturning at a higher density than the peak of the surface water mass transformation [39,41,47].

## 3. Three-dimensional structure of the mean overturning

As waters circulate cyclonically around the subpolar gyre, they are cooled progressively by air–sea fluxes and freshened by mixing with shelf waters. Deep convection, mixing of waters between the surface and depth, which results in a thick layer of homogeneous fluid, occurs at the termini of this densification. In the subpolar gyre, deep convection occurs in the Labrador Sea and intermittently in the Irminger Sea, and the waters formed in these basins are generally referred to as Upper North Atlantic Deep Water (UNADW). Rather than circulating cyclonically around the subpolar gyre, some waters enter the Nordic Seas east of Iceland [48]. Progressive densification occurs as Atlantic waters circulate around the Nordic Seas [49–52], feeding deep convection sites, primarily in the Greenland Sea. Dense waters which are formed in the Nordic Seas and enter the North Atlantic by flowing over the Greenland–Scotland Ridge are generally referred to as lower North Atlantic deep water (LNADW) or OWs. In this section, we discuss the dynamics and thermodynamics of water mass transformation, deep convection and export of waters from convective regions. Water mass transformation, deep convection and the interaction between boundary currents and the convective interior are similar across regions, but the export of dense waters from the marginal sea differs between the Labrador/Irminger Sea and the Nordic Seas.

**Figure 3.**
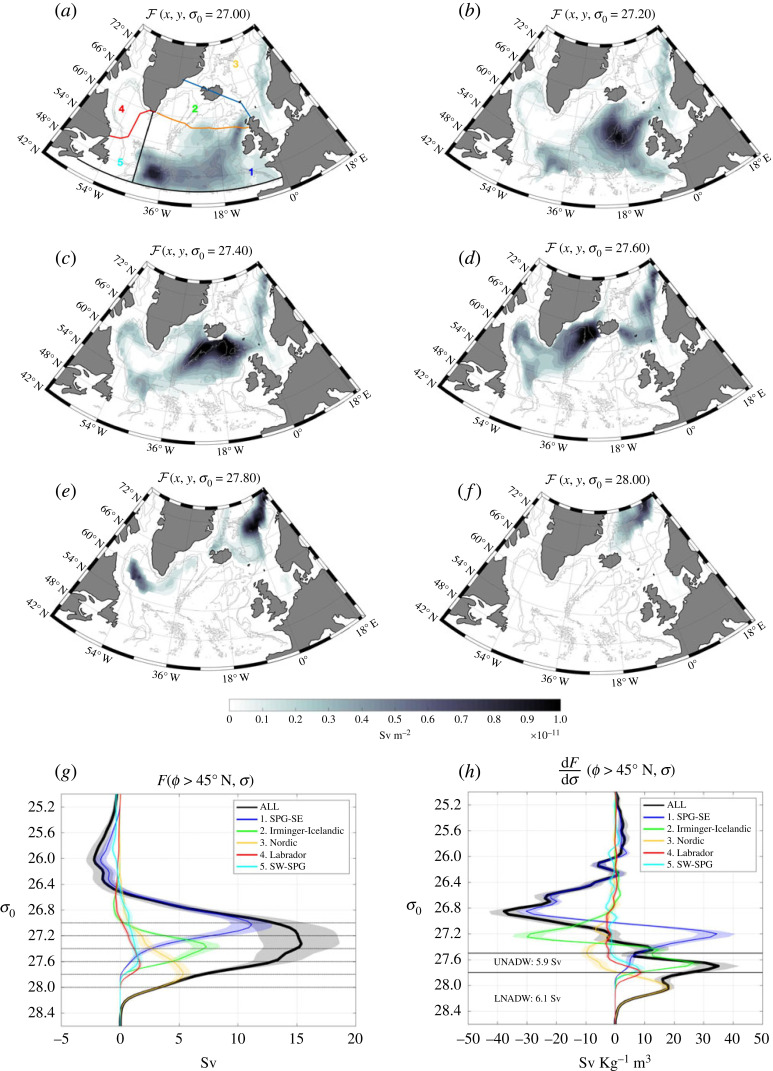
(*a*–*f*) Maps of surface water mass transformation, F(x,y,σ) (see equation (2.6)), at different $\sigma_{0}$ surfaces within the subpolar North Atlantic and Nordic Seas. (*a*) The OSNAP East and OSNAP West sections are indicated in orange and red, respectively, and the Greenland–Scotland Ridge is indicated in blue. (*g*) Surface water mass transformation between $45^{\circ }\;\text{N}$ and $80^{\circ }\;\text{N}$, $F(45^{\circ }\;\text{N}<\phi <80^{\circ }\;\text{N},\sigma )$ (*h*). The convergence in density space of surface water mass transformation, $-\partial F/\partial \sigma (45^{\circ }\;\text{N}<\phi <80^{\circ }\;\text{N},\sigma )$. (*g*,*h*) The total is shown in black and the contribution from different regions is shown in the coloured lines (regions are shown in (*a*)). Shading indicates the ensemble standard deviation. (*h*) The surface water mass formation is indicated for UNADW ($27.5<\sigma_{0}<27.80\;{\text{ kg m}}^{-3}$) and LNADW ($\sigma_{0}>27.80{\text{ kg m}}^{-3}$). For all results presented (panels *a*–*h*), surface density computed from the EN4.2.2 ocean analysis [57] with the Gouretski & Reseghetti bias corrections [58] applied is paired with distinct buoyancy fluxes estimates NCEP2 [59], ERA5 [60] and heat flux from CERES [61] (salt fluxes from NCEP2 are used for the latter). The average is computed over the period 2002–2018.

### (a) Geography of water mass transformation

In this section, we use surface water mass transformation maps (equation (2.6)) and regional analysis of water mass transformation to understand the main locations of the mean surface water mass transformation. Surface waters are progressively densified by surface buoyancy loss as they circulate cyclonically around the subpolar gyre [34,39,41,53–56], first along the North Atlantic Current (NAC) and in the southeastern subpolar gyre (figure 3*a*, purple line in figure 3*g*), then in the Iceland basin (figure 3*b*,*c*, green line in figure 3*g*), then the Irminger Sea (figure 3*d*, green line in figure 3*g*) and finally within the Labrador Sea (figure 3*d*,*e*, cyan line in figure 3*g*). Water mass transformation in the densest classes occurs in the Nordic Seas (figure 3*e*,*f*, yellow line in figure 3*g*). Prior work showing maps of water mass transformation based on observations (Fig. 4 in [53]) shows similar patterns, although they find smaller water mass transformation within the Labrador Sea in the OW density range. The presence of water mass transformation in the OW density class within the Labrador Sea appears related to outcropping of unrealistically dense waters in EN4 and may not be present in analyses with other data products. Water mass transformation maps in a high-resolution model also show similar patterns of transformation (Fig. 13 in [39]), although the amplitude of the water mass transformation in the Labrador Sea is overestimated in this model due to model biases.

The importance of air–sea buoyancy forcing in creating/destroying water masses in different regions can be seen from the convergence of the surface water mass transformation in density space (figure 3*h*). Light waters of subtropical origin are destroyed and converted to denser waters in the southeastern subpolar gyre (blue line). Nearly all the waters formed in the southeastern subpolar gyre are destroyed within the Irminger Sea/Iceland basin, being converted to denser UNADW water (green line). NADW is converted to even denser waters in the Labrador Sea (red line) and the southwestern subpolar gyre (cyan line). Yet, much of the peak in water mass formation (black line, peak at $\sigma =27.7{\text{ kg m}}^{-3}$), classically referred to as Labrador Sea Water (LSW), is related to water mass formation in the Irminger/Iceland basin. In this observational estimate, surface water mass formation creates 5.9 Sv of UNADW ($27.5<\sigma_{0}<27.80{\text{ kg m}}^{-3}$) and 6.1 Sv of LNADW ($\sigma_{0}>27.80{\text{ kg m}}^{-3}$). The magnitude of the water mass transformation/formation in each region and whether there is a single peak or a separate peaks in subpolar mode waters and UNADW densities exhibit differences between and among observations [47,62] and models [46,55] and is sensitive to model resolution [63]. For example, [56] finds much stronger water mass formation in the Labrador Sea than observational estimates presented here indicate (see Fig. 3*a*,*b* in [56]).

There has recently been much discussion in the literature about the relative importance of the eastern basin and western basin in the transformation of waters into the lower limb of the AMOC [16,30,43,56,64]. Over the period August 2014 to May 2016, about 2 Sv is transformed into the lower limb in the Labrador Sea (across OSNAP West), while more than 6 Sv is transformed in the eastern basin between OSNAP East and the Greenland–Scotland Ridge [33,43]. An additional 6.6 Sv of water is transformed into the lower limb in the Nordic Seas [65–67], contributing to the total of about 13 Sv of lower limb water crossing OSNAP East [43]. A recent analysis of the extended OSNAP time series (August 2014 to June 2020) yields an overturning of 3 Sv across OSNAP West and 16.3 Sv across OSNAP East, a slight increase in the mean for both, though almost entirely within the bounds of uncertainty [33]. The small surface water mass transformation in the Labrador basin is consistent with the progressive nature of the densification; surface waters are already very dense when they enter the Labrador Sea, having already been cooled in the Iceland/Irminger basins [66]. In addition, Labrador Sea waters are freshened by mixing with cold, fresh shelf waters sourced from Arctic outflows and meltwater from Greenland [68]. As salinity has increasing importance to density at the low temperatures, this freshening is able to compensate much of the cooling experienced by waters transiting the Labrador Sea, leading to a small amount of surface water mass transformation and overturning in density coordinates in the Labrador Sea [32].

Climate models, particularly those with coarse resolution, often overestimate the surface water mass transformation and overturning in the Labrador (western) basin [69]. Models generally produce OWs that are too light due to excessive mixing with ambient (lighter) waters [70]. As a result, in the Labrador Sea, the mid-depth stratification is too weak, the mixed layer depths are too deep [71–73], the volume of LSW produced is too large [69] and the overturning is too strong. Salinity biases in models may also contribute to the too-strong overturning in density space. Many models lack the cold, low salinity shelf water from the Arctic [39] and without these compensating freshwater transports the overturning in density space may resemble the stronger overturning in temperature space [32].

### (b) Dynamics of deep convection

While buoyancy loss and water mass transformation takes place over large areas of the ocean (§3a), deep convection occurs in relatively isolated regions (e.g. the Labrador Sea, Nordic Seas and intermittently in the Irminger Sea [74–76]). It is in these regions of deep convection that large volumes of the subsurface ocean are exposed to the atmosphere. As a result, these regions are essential for the uptake and sequestration of heat, carbon and oxygen by the ocean [77].

Convective regions share features which predispose them to intermittent, deep reaching convection. The stratification must be weak, and weakly stratified waters must be brought towards the surface to be exposed to atmospheric forcing [75,78]. Ekman upwelling in the subpolar gyre results in the doming of isopycnals towards the surface, exposing weakly stratified waters to atmospheric forcing. In addition, there must be strong atmospheric forcing causing densification of surface waters (cooling or brine rejection resulting from sea ice formation). Thus, open ocean regions adjacent to boundaries are favoured, where cold, dry winds from adjacent continents blow over the water and extract heat.

Convection is an intermittent process in both space and time and involves the interaction of processes at a variety of scales (see comprehensive reviews by [75,79]). The convective process can be thought of as occurring in three phases: (1) preconditioning, (2) deep convection and (3) lateral exchanges, although the last two phases often occur concurrently [75,80]. During preconditioning, buoyancy loss erodes the stratification of the cyclonic dome over an area of a few hundred kilometres, exposing weakly stratified waters. Deep convection occurs in localized, small-scale plumes in which properties are homogenized in the vertical (or in the case of slantwise convection, properties are mixed along isopycnals [81–83]). The plumes have small horizontal scales (order 1 km), but many plumes acting in concert can form a deep mixed patch with a size of tens of kilometres to greater than 100 km. The final stage is lateral exchange by eddies, which is important for restratification of the convective patch [20,84–87].

### (c) Export of dense waters from a marginal sea

**Figure 4.**
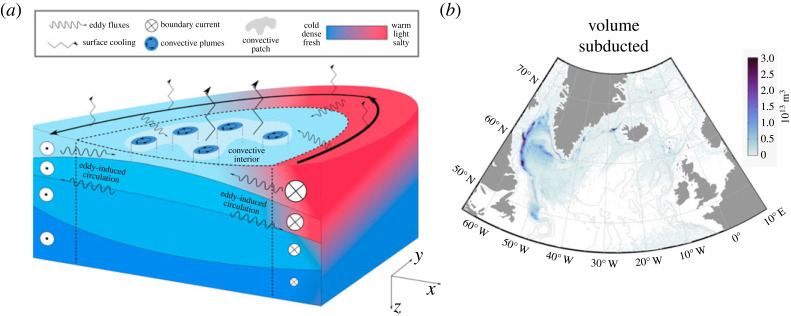
(*a*) Schematic of a marginal sea. The basin interior is defined by closed geostrophic contours, and the boundary current region is defined by open geostrophic contours. The boundary current is less dense than the interior. Exchange between the interior and boundary current is driven by baroclinic eddies. (*b*) Volume of NADW subducted (for the final time prior to be exported to the subtropics) at each grid point over full time of simulation (1958–2015) from back trajectories initialized in NADW layer in historically forced NEMO run (1958–2015). Light grey lines correspond to bathymetric contours at depths of approximately 500, 150, 3000 and 3900 m. Adapted from [70]. Note, this model has excessive mixing of OWs, too deep mixed layer depths in the Labrador Sea, and overproduces LSW. As a result, most water subducted in the Nordic Seas is reentrained within the mixed layer in the Labrador Sea, explaining why there is very little water that is subducted for the last time in the Nordic Seas. This is clearly a model deficiency, so this map is better interpreted as a map of subduction of UNADW. As nearly all the UNADW formed in the Irminger/Icelandic basins passes through the Labrador Sea, one might expect the final subduction of UNADW to occur in the Labrador Sea, as we see here. Yet, it remains unclear whether the pattern of subduction would change in a model with more realistic LSW formation.

The eddy-driven interaction between the convective interior and the boundary current plays a key role in setting the final properties of dense water masses and the strength of the overturning [88–90]. Marginal seas, such as the Labrador Sea, can be idealized as regions with closed geostrophic contours in the interior (where deep convection occurs) and open geostrophic contours around the basin perimeter [87]. In this framework, any waters entering and exiting the marginal sea must do so in the boundary current, and, thus, the overturning is equivalent to the densification of the boundary current waters. The boundary current waters are lighter (warmer) at the surface and also more stratified than the interior waters. As a result, the boundary current and the interior share isopycnals at depth, which is a key to the eddy-driven interaction between these regions (figure 4*a*).

There are two means by which the boundary current waters can be densified: directly within the boundary current and through interaction with interior waters. Waters within the boundary current can be directly densified as the boundary current cools due to surface heat loss and diapycnal eddy heat fluxes. Waters also can be densified in the interior and then exchanged along isopycnals between the boundary current and the interior by eddies [88–92]. The eddy-driven circulation, which acts to slump the steep isopycnal slopes between the interior and the boundary current, transports light waters out of the boundary current and dense waters into the boundary current [91] (figure 4*a*). As a result, as compared to the inflowing boundary current, the outflowing boundary current is not only cooler at the surface, it is more barotropic (less shear in both depth and density space) [88–91]. The barotropization of the boundary current can be seen in observations: while the northward West Greenland Current in the east of the Labrador Sea is strongly sheared, the southward Labrador Current in the west of the Labrador Sea displays less vertical shear [31,93].

Lower limb waters which are transformed in the boundary current have different densities and export timescales than those transformed in the interior. Export of dense waters formed within the boundary current is rapid, but it only involves the lighter waters in the lower limb, as the boundary current still remains relatively warm and stratified along the basin perimeter (figure 4*a*). Since the water in the interior is colder and less stratified, waters formed in the interior are denser. The export timescale of these waters is longer than those formed within the boundary current because these waters must be transported into the boundary current by eddies to exit the marginal sea [94,95]. Thus, dense waters will have a large range of residence timescales in a marginal sea, depending on their pathways, and these pathways are controlled by ocean dynamics.

While the interaction between the convective interior and the boundary current is a common feature of convective basins, the export of waters from the Nordic Seas is strongly constrained by local bathymetry. Dense waters formed in the Nordic Seas must flow over the Greenland–Scotland Ridge [96]; as mentioned earlier, these waters are referred to as OWs or LNADW. As the dense boundary current flows over the sill, it mixes with the lighter ambient Atlantic waters, resulting in significant entrainment and modification of water mass properties. Denmark Strait overflow water (DSOW), which exits between Greenland and Iceland, is the coldest and densest OW ($\sigma_{0}>27.99{\text{ kg m}}^{-3}$). Iceland–Scotland overflow water (ISOW), which exits between Iceland and Scotland, is warmer, saltier and less dense ($\sigma_{0}=27.80-27.88\;{\text{ kg m}}^{-3}$) as a result of the entrainment of saltier subpolar mode waters.

### (d) Spreading of NADW and export to the subtropics

Most UNADW formed in the Irminger and Icelandic basin, as well as DSOW, enters the Labrador Sea, and these waters can be seen in both the boundary currents and interior (see [97–101] for UNADW pathways and [9,102] for DSOW pathways). For example, [103] shows that the majority of UNADW passes through the Labrador Sea prior to being exported to the subtropics. In the Labrador Sea, UNADW is densified, cooled and freshened. In addition, backward-in-time trajectories launched in the NADW layer in an eddy-permitting model show that the Labrador Sea is the main location where UNADW undergoes its final subduction (loss from the mixed layer) before being exported to the subtropics (figure 4*b*) [70,104], although the importance of the Labrador Sea for subduction may be exaggerated due to excessive LSW formation in this model. Therefore, despite the large water mass transformation in the Iceland and Irminger Basins, these waters are further modified within the mixed layer in the Labrador Sea prior to subduction and export to the subtropics. In contrast to UNADW and DSOW, ISOW primarily spreads east of the Mid-Atlantic Ridge [9,105].

At the exit of the Labrador Sea, the DWBC transports about 30 Sv of NADW southward, which is about twice the overturning strength at this latitude [106]. This implies strong recirculation of NADW within the subpolar gyre, primarily the deep cyclonic circulation in the western subpolar gyre [107], but also in narrow recirculations adjacent to the boundary current [108,109]. The deep reaching NAC carries a substantial amount of NADW recirculating within the subpolar gyre [110].

**Figure 5.**
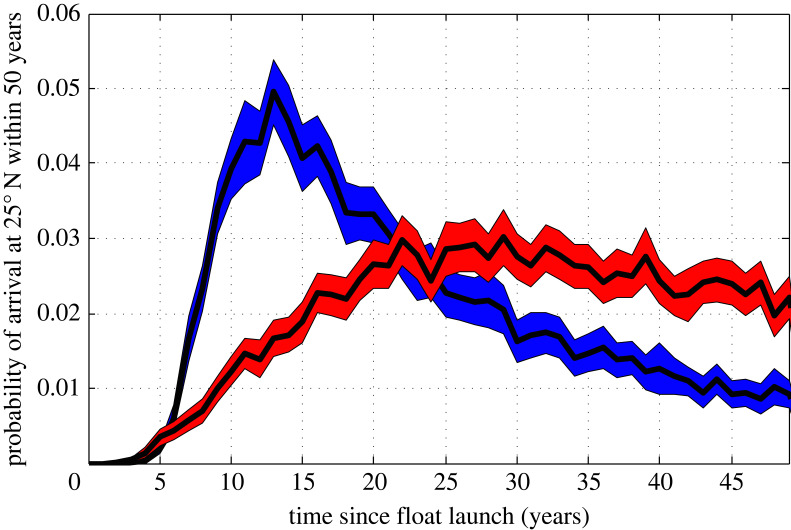
Transit time distribution of UNADW (red) and OW (blue) floats launched at $53^{\circ }\;\text{N}$ within the DWBC arrival at $25^{\circ }\;\text{N}$. Only the portion of floats that reached $25^{\circ }\;\text{N}$ within 50 years were used to construct the transit time distribution. Shading indicates one standard deviation of the transit time distribution calculated from a Monte Carlo simulation. Adapted from [102].

Recirculation of NADW within the vigorous subpolar gyre, eddy-driven recirculations and mixing with older waters (1) lengthen the pathways to the subtropics and (2) result in a multitude of possible pathways. While the fastest pathway between the subpolar gyre and the subtropics is the DWBC [9,98,111], most NADW passes through the interior of the basin prior to export to the subtropics [112–114]. The mean age of NADW entering the subtropics is on the order of decades [98,102,118]. As a result of the multitude of pathways, NADW at any particular location (e.g. entering the subtropics) has a wide range of ages, as measured by the time since the water was at the surface (figure 5). The range of timescales between the source (e.g. the Labrador Sea) and a location of interest (e.g. the subtropical–subpolar gyre boundary) can be quantified in terms of a transit-time distribution [102,119–122]. The mean residence timescale and the range of residence timescales of NADW have strong implications for understanding variations in the AMOC, as will be discussed in §4.

Aging of tracers and ejection of NADW from the DWBC into the interior does not occur continuously along the pathway of the DWBC, but rather abruptly within the subtropical–subpolar gyre transition zone [98,112,117,123]. Here, tracer ages in the DWBC increase the most rapidly and the fraction of young waters decreases most abruptly, indicating strong mixing with older NADW (Fig. 3 of [98]). Upon reaching the gyre boundary, a significant portion of the DWBC is recirculated within the subpolar gyre and thus does not enter the subtropics. In addition, water in the DWBC is injected into the interior due to inertial separation near bathymetric curvature and steepening [117] and eddy-driven recirculation gyres generated by the instabilities of the Gulf Stream/NAC system [114–117]. Floats launched in the UNADW layer of the DWBC at $50^{\circ }\;\text{N}$ escape the DWBC primarily between the Flemish Cap and just upstream of the Tail of the Grand Banks (see Fig. 2*b* of [112]). For particles released in OWs east of Greenland, the DWBC is the dominant pathway at $50^{\circ }\;\text{N}$, but the interior pathway accounts for more than 85% of particles that reach by $40^{\circ }\;\text{N}$ [9].

## 4. Variability of the AMOC

In this section, we discuss the extent to which variability of the overturning in density coordinates can (or cannot) be related to upstream variations in water mass transformation. We do not focus on tracking changes in properties of NADW along the DWBC [124–126], which will be covered in another article in this issue. We first focus on the relationship between variations in water mass transformation and local overturning; then we focus on the overturning anomalies that may be communicated meridionally to the subtropical gyre. Our focus is mostly on UNADW rather than OWs, as observations suggest that the overturning in the OW density range does not have strong variability [127,128]. A more general discussion of AMOC variability on intraannual to multi-decadal timescales may be found in [13,129].

### (a) Variations of the AMOC in a marginal sea

Firstly, we discuss the relationship between variations in water mass transformation and local export of dense waters, meaning export across a latitude only a short distance downstream (equatorward) of regions of water mass transformation into the lower limb. This situation applies to export out of the Labrador Sea (equivalently OSNAP West) or across OSNAP East, which is just downstream of UNADW formation regions.

**Figure 6.**
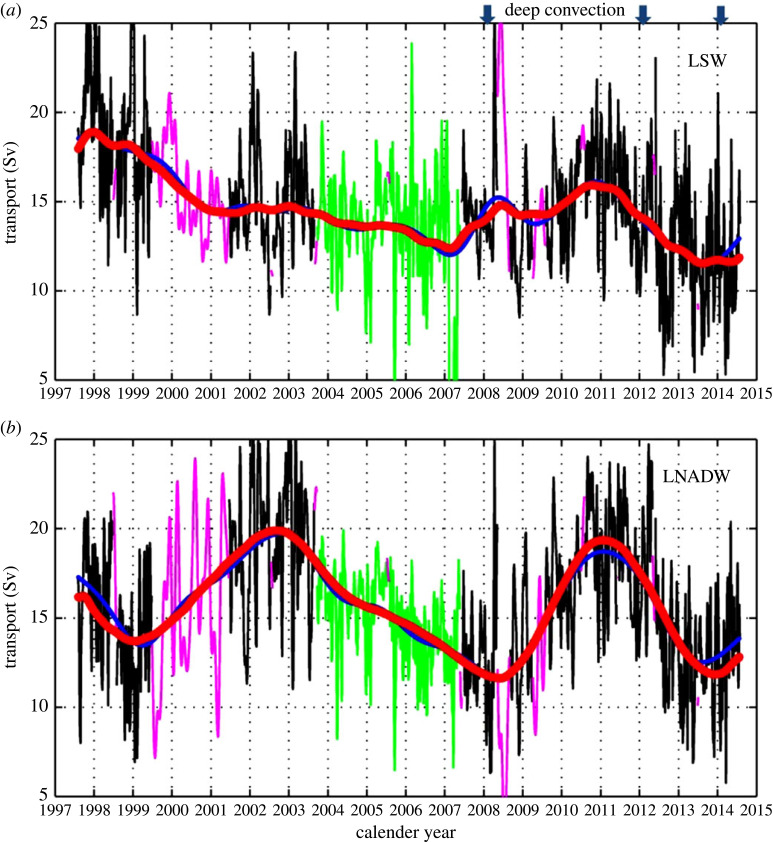
Time series of transport out of the Labrador Sea at $53^{\circ }\;\text{N}$ for (*a*) UNADW and (*b*) LNADW. Black lines are for periods of full array coverage; green lines for periods with reduced coverage but with central mooring K9 in place, and magenta lines are regions where gaps have been filled by a statistical analysis. Periods with enhanced deep convection in the Labador Sea are marked at the top of (*a*). Figure is adapted from [106].

Historically, observations have attempted to relate changes in deep convection to changes in overturning. Observations show strong variability in deep convection in the Labrador Sea [130–132] and Irminger Sea [133], as well as variations in properties of UNADW [78,134]. However, these changes in convection do not, in general, result in changes in export from the Labrador Sea [7,8,31]. For example, despite strong changes in deep convection over observational periods, the DWBC at $53^{\circ }\;\text{N}$ remained relatively steady [4,106,134,135]; modest changes in export from the Labrador Sea do not bear an obvious relationship to changes in deep convection (figure 6*a*). Similarly, the volume transport of OW at the Greenland–Scotland Ridge is observed to be quite steady [127,128], despite variations in convection [136] and water mass properties [137] in the Nordic Seas.

Yet, we now know that surface water mass transformation, deep convection and overturning in density space are distinct processes (§3) and are not expected to have a one-to-one correspondence [8,138]. As a result, when examining how variations in production of dense waters may (or may not) lead to variations in overturning, a better approach is to use the Walin framework [28], which mathematically relates water mass transformation, volume changes between isopycnals and local export of dense waters (equation (2.4)). The relationship between these terms depends on the timescale of variability, specifically whether it is shorter or longer than the residence timescale of waters in the marginal sea. Residence timescales of UNADW in the Labrador Sea are generally thought to be several years [139], although UNADW formed in the boundary current is exported rapidly and UNADW formed in the interior is exported more slowly, as it first needs to reach the boundary current through eddy exchange [95,104].

If water mass transformation varies on timescales shorter than the residence timescale of waters in the marginal sea, then there is a local balance between water mass transformation and volume changes between isopycnals. Variability in the export of dense waters (overturning in density space) is much less than the variability of the water mass transformation, indicating that the marginal sea buffers changes in air–sea heat fluxes (e.g. surface water mass transformation) from being communicated to the rest of the ocean [87]. As a result, high-frequency atmospheric variability is expected to alter the volume and properties of dense waters, but these variations will not cause significant changes in export of dense waters from a marginal sea. It is well established that seasonal variations in water mass transformation primarily result in changes in volume between isopycnals (see Fig. in 3 [140]), but at OSNAP, the large seasonal changes in transformation do result in modest seasonal changes in export [33].

In contrast, if water mass transformation varies on timescales longer than the residence timescale of waters in the marginal sea, these changes are expected to result in changes in overturning (export of dense waters) [87]. Thus, we expect that persistent changes in water mass transformation due to, for example, anthropogenic climate change will result in changes in export.

Here, we examine the relationship between annual means of surface water mass transformation into the lower limb ($F(\sigma_{\mathrm{MOC}})$), diffusive transformation into the lower limb ($G_{D}(\sigma_{\mathrm{MOC}})$), volume changes of the lower limb and export of dense waters ($\Psi (\sigma_{\mathrm{MOC}})$) between OSNAP East/OSNAP West and the Greenland–Scotland Ridge/Davis Strait in ECCO v4 over the period 1992–2017. Between OSNAP East and the Greenland–Scotland Ridge, annual variations in water mass transformation into the lower limb are mainly balanced by volume changes of the lower limb, with generally smaller variations in export of dense waters, as found by [141]. Between OSNAP West and the Davis Strait, the relationship is more complicated, in particular water mass transformation due to mixing is substantial. Yet, the total water mass transformation ($F+G_{D}$) is mostly balanced by volume changes rather than changes in export. In ECCOv4, interannual variations in water mass transformation are larger across OSNAP West than OSNAP East, which is likely related to model biases in the Labrador Sea, although both models [56] and reanalysis products [141] suggest that the relative importance of OSNAP West increases with increasing timescale. Repeating this analysis with a high-resolution ocean state estimate, such as the Arctic Subpolar Atlantic State Estimate (ASTE, [142]), would be informative. Yet, the conclusion that interannual changes in volume between isopycnals are important and variations in water mass transformation cannot be directly connected to interannual changes in overturning is supported by observation analyses [43] and is likely robust.

Model biases may impact the relationship between water mass transformation and export. Models, particularly coarse-resolution models, tend to overproduce LSW in the mean (see §3a), and variability of LSW formation is also larger than observed [39,69,70]. Comparing volumes of LSW between models and observations also suggests that models either flush LSW out of the Labrador Sea too rapidly or low potential vorticity waters are destroyed too quickly because models are overly diffusive [69]. As a result of overproduction of LSW and potentially too rapid export, the relationship between LSW formation and export appears to be unrealistically strong in some models. However, the overly diffusive nature of coarse-resolution models can also potentially lead to unrealistically weak relationships between water mass transformation and export. Studying such relationships in OSNAP observations and high-resolution models [63,143–145] and ocean reanalyses [141] will help clarify relationships between water mass formation and export.

### (b) Communication of AMOC variations to the subtropics

**Figure 7.**
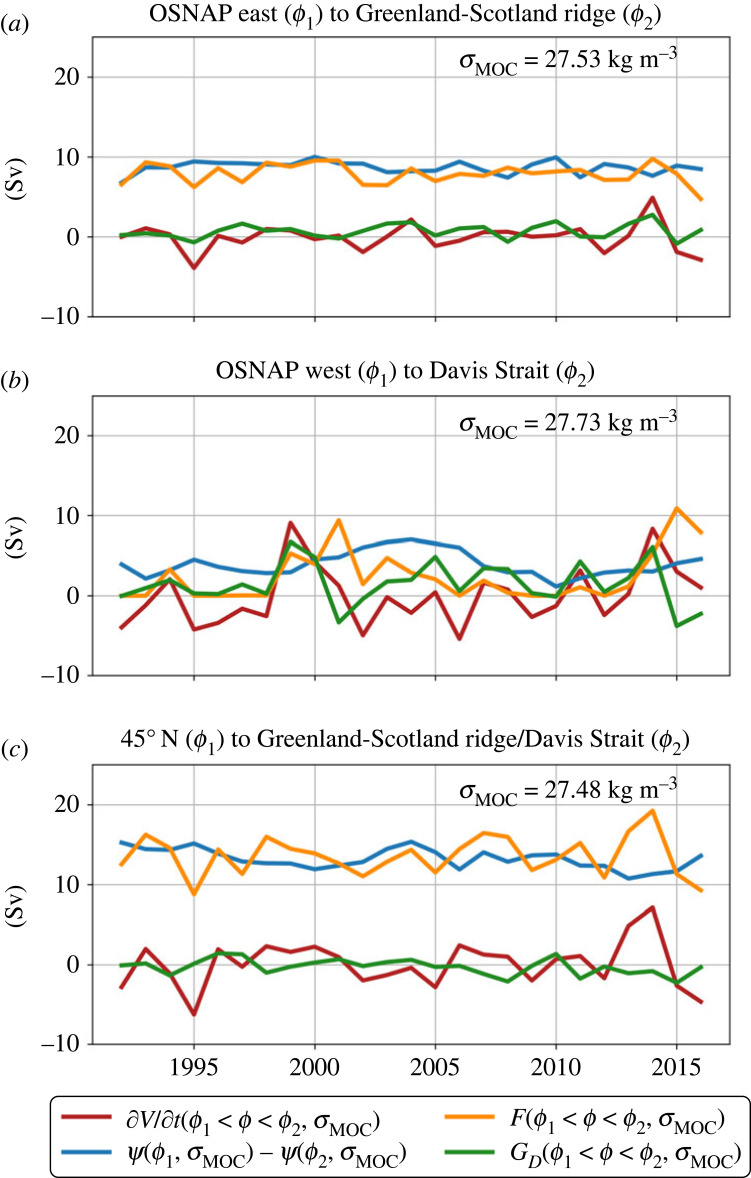
(*a*) Annual mean (June through May, to keep winter season contiguous) density/volume budget of the lower layer (below $\sigma_{\mathrm{MOC}}$) for (*a*) OSNAP East to the Greenland–Scotland Ridge, (*b*) OSNAP West to Davis Strait and (*c*) $45^{\circ }\;\text{N}$ to the Greenland–Scotland Ridge/Davis Strait from Estimating the Circulation and Climate of the Ocean version 4 (ECCO v4) [10]. The plot shows the surface water mass transformation, $F(\phi_{1}<\phi <\phi_{2},\sigma_{\mathrm{MOC}})$ (orange line); the overturning, $\Psi (\phi_{1},\sigma_{\mathrm{MOC}})-\Psi (\phi_{2},\sigma_{\mathrm{MOC}})$ (blue line) and the volume change of the layer below isopycnal $\sigma_{\mathrm{MOC}}$, $\partial V/\partial t(\phi_{1}<\phi <\phi_{2},\sigma_{\mathrm{MOC}})$ (red line). The green line indicates the residual, which is interpreted as the water mass transformation due to mixing $G_{D}(\phi_{1}<\phi <\phi_{2},\sigma_{\mathrm{MOC}})$. $\phi_{1}$ and $\phi_{2}$ are the specific boundaries specified in (*a*–*c*). The values of $\sigma_{\mathrm{MOC}}$ for each region are listed in the top right of each figure.

Now we discuss AMOC variations which reach the subtropics, and how they may (or may not) be related to changes in upstream water mass transformation. As in the previous section, we will utilize the Walin framework [28] (equation (2.4)) and note that the relationship between water mass transformation, volume changes between isopycnals and local export of dense water depends on the timescale of variability, specifically whether it is shorter or longer than the residence timescale of NADW in the subpolar gyre. As residence timescales of NADW in the subpolar gyre are on the order of decades [98,102,118] (figure 5), changes in rates of water mass transformation on timescales shorter than this residence time likely lead to changes of volumes of water masses, rather than significant changes in export to the subtropics [47,146,147]. Indeed, a quantification of the volume budget of the lower limb waters between $45^{\circ }\;\text{N}$ and the Greenland–Scotland Ridge/Davis Strait using ECCO v4 demonstrates that variations in the surface water mass transformation into the lower limb, $F(\sigma_{\mathrm{MOC}}$), are highly correlated with volume changes of the lower limb, although variations in the water mass transformation due to mixing, $G_{D}(\sigma_{\mathrm{MOC}})$, cannot entirely be neglected (figure 7*c*). Variations in export, $\Psi (\sigma_{\mathrm{MOC}})$, are smaller than both surface water mass transformation and volume changes.

Given that in the mean, the total water mass transformation must be balanced by export, one might expect that one could find a relationship between the variability of water mass transformation and export on some timescale longer than the residence timescale. However, the large range of residence timescales of NADW entering the subtropics (figure 5) likely obscures finding such a relationship because waters entering the subtropics are a mixture of waters formed at different times, during different conditions. Thus, only large and persistent changes in water mass transformation have the potential to be communicated to the subtropics [69,118]. For example, if climate change leads to a continuous warming of surface waters and a reduction of water mass transformation into the lower limb, one would expect this signal to eventually reach the subtropics. On the other hand, high-frequency changes in water mass transformation, for example those driven by high-frequency atmospheric variations, are not expected to be communicated to the subtropics.

A number of studies have sought to link decadal variations in surface water mass transformation ($F(\sigma_{\mathrm{MOC}})$) to overturning ($\Psi (\sigma_{\mathrm{MOC}})$) at a range of latitudes within the subpolar and subtropical gyre using observations [47], reanalysis [148,149], forced ocean models [55,150] and coupled climate models [148,151]. Consistent with the larger variance of $F(\sigma_{\mathrm{MOC}})$ compared to $\Psi (\sigma_{\mathrm{MOC}})$ and the long residence time of waters in the subpolar gyre, $F(\sigma_{\mathrm{MOC}})$ is accumulated over time period $\tau_{A}$ before being compared to $\Psi (\sigma_{\mathrm{MOC}})$. The parameter $\tau_{A}$ is chosen to obtain the best correlation between $\bar{F(\sigma_{\mathrm{MOC}})}$ (overbar denote time average over period $\tau_{A}$) and $\Psi (\sigma_{\mathrm{MOC}})$, and $\tau_{A}$ is found to be longer for lower latitudes, as expected since both the mean age and the range of ages of NADW increases moving southward. The strength of the relationship between $\bar{F(\sigma_{\mathrm{MOC}})}$ and $\Psi (\sigma_{\mathrm{MOC}})$ is found to depend on the time period analysed, with stronger correlations found for time periods with large variations of $\Psi (\sigma_{\mathrm{MOC}})$ [150]. This confirms that strong relationships between surface water mass formation and export exist only for strong and persistent anomalies in surface water mass transformation.

Models, particularly those with coarse resolution, may be missing the complex array of processes that lead to spreading of UNADW. As a result, downstream AMOC variability may be too tightly tied to upstream variations in source waters. Model experiments suggest that large-scale, decadal AMOC variability in the North Atlantic primarily results from buoyancy forcing over subpolar regions [–27,152,154] related to the North Atlantic Oscillation (NAO) [154–159]. Models have particularly emphasized buoyancy forcing over the Labrador Sea as being important for AMOC variations [25,160]. However, this strong relationship is certainly influenced by the tendency of models to overproduce LSW and overestimate LSW variability (see §§2b and 4(a)). A recent intermodel comparison [161] demonstrates that the relationship between changes in the Labrador Sea and the subtropical AMOC is model dependent, and comparison with observations suggests that models with weaker links between the Labrador Sea and the subtropical AMOC are more realistic.

### (c) AMOC variability generated in the subtropical–subpolar transition zone

Due to the multitude of pathways by which NADW may reach the subtropics and, thus, the large range in transit times to the subtropics, any source water variations are expected to be attenuated greatly. As a result, one must consider the possibility that variations in export of dense waters to the subtropics are controlled by processes other than source water variations.

An obvious possibility is that changes in export of NADW across the gyre boundary are related to interactions between NADW and vigorous upper ocean currents. Within the subtropical–subpolar gyre transition zone, the southward flowing DWBC interacts with the northward flowing NAC. Changes in the strength or the path of upper ocean currents, such as the NAC, may influence the pathway and volume transport of NADW across the gyre boundary. Using several different ocean models, Zou *et al.* [162] suggest that interannual variations of a large-scale mode of AMOC variability are driven by changes in westerly winds along the subtropical–subpolar gyre boundary. Jamet *et al.* [163] show that near the gyre boundary, atmospheric forcing can cause AMOC variability on decadal timescales, and these anomalies interact with the decadal AMOC variations originating from the subpolar gyre. Polo *et al.* [164] also find decadal wind-driven AMOC signals at $26^{\circ }\;\text{N}$, which are related to both Rossby wave propagation and decadal modulation of buoyancy-forced AMOC signals. From an analysis of 17 years of boundary current transports at the exit of the Labrador Sea at $53^{\circ }\;\text{N}$, [106] shows that there are quasi-decadal fluctuations in the LNADW layer that appear to be in phase with NAO-modulated wind fluctuations (figure 6*b*). Modelling results from [165] show that a dynamical response of the NAC to surface density changes originating in the Labrador Sea can result in changes within the LNADW layer.

Eddies and instabilities of vigorous upper ocean currents can lead to changes in transport and pathways of NADW [110]. Interactions between NADW and the upper ocean were observed on several hydrography snapshots at $47^{\circ }$N, where deep-reaching eddies shed from the NAC into the DWBC were found to significantly alter the volume transport and the thermohaline properties of the NADW [110]. The interaction of the upper ocean circulation and NADW at the subtopical-subpolar transition zone is a topic that may soon be addressed by ongoing and planned observational campaigns in this region.

### (d) Local wind-driven AMOC variations in the subtropics

The AMOC exhibits large variability on intraannual and seasonal timescales (order 100% of its mean value) and much smaller variability on interannual to decadal timescales (order a few Sv). In the subtropics, seasonal and intraannual AMOC variability is primarily the local response to wind variability [166–169], both Ekman transports and wind-induced heaving of isopycnals. The spatial scale of wind-induced AMOC variations is set by that of wind stress variations and thus reflects the scale of dominant modes of atmospheric variability, such as the NAO. As mentioned earlier, as a result of gyre-specific wind-forcing anomalies, the AMOC is not coherent between the subtropical and subpolar gyres on interannual timescales [170–173]. The AMOC in the subtropical gyre is dominated by high-frequency, wind-driven variability, while AMOC variations in the subpolar gyre are generally lower frequency, and both wind and buoyancy forcing play a role [27,152,174,175].

**Figure 8.**
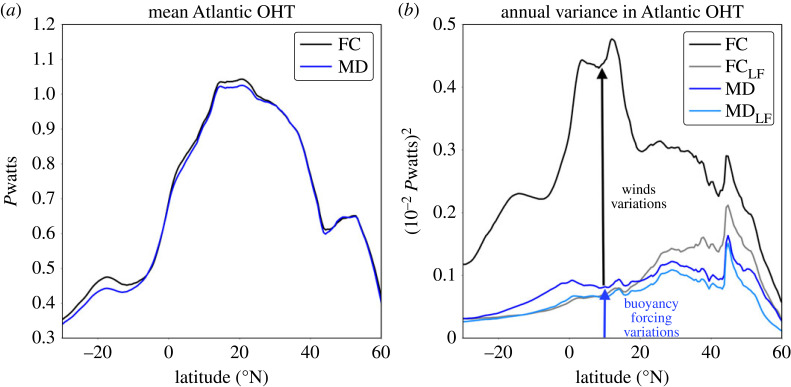
(*a*) Annual mean northward Atlantic oceanic heat transport (OHT) due to Eulerian-mean advection from fully coupled (FC, black line) and mechanically decoupled (MD, blue line) versions of CESM2. (*b*) Variance of annual Atlantic Ocean OHT in the FC (black line) and MD (blue line) simulations and the 10 year low-pass filtered variances of Atlantic OHT in FC (${\text{FC}}_{\text{LF}}$, grey line) and MD (${\text{MD}}_{\text{LF}}$, light blue line). Units are ${\left(10^{-2}\;\mathrm{PW}\right)}^{2}$. Figure produced by Kay McMonigal. Similar plots for CESM1 are shown in [174].

An obvious question is whether these wind-forced AMOC variations merely impart noise upon the buoyancy-forced signals or whether the wind-forced variations modify AMOC variability in a more consequential way, perhaps by destroying buoyancy-forced variations. Here, we address this question by comparing a fully coupled model (ocean forced by wind and buoyancy forcing) with a coupled model in which the ocean model is forced by the seasonal climatology of wind stress, but thermodynamic forcing remains unaltered, referred to as the mechanically decoupled model [174,176]. The mean Atlantic OHT in the mechanically decoupled model is nearly identical to the fully coupled model, as the mean ocean forcing is unaltered (figure 8*a*). The difference in variability between the two models is indicative of the role of variations in wind forcing. One can see that wind-forcing imparts strong variability in Atlantic OHT in the tropics/subtropics, but buoyancy forcing becomes more important for OHT variations in the subpolar North Atlantic (figure 8*b*). Low-pass filtering significantly reduces the variance of Atlantic OHT in the fully coupled model (figure 8*b*), suggesting most of the wind-induced Atlantic OHT variations are high-frequency noise superimposed on the buoyancy-forced signal. However, this model is a coarse-resolution climate model that exhibits a too strong overturning in the Labrador Sea [56,69] and likely also a too strong relationship between upstream water mass transformation and the downstream AMOC [161]. It may be easier to remove the impact of winds upon a strong buoyancy-induced signal (as seen in models) compared to a more muted one (as is suggested by observations). Repeating this type of experiment in a high-resolution model may yield different results.

### (e) Discrepancies between overturning diagnostics in observations and models

The nature of AMOC observations, which are limited to a few latitudes and a decade or two at most, makes it hard to observe relationships between variations in upstream water mass transformation and overturning, even if there are robust relationships between these quantities, as models suggest.

Modelling studies which suggest a dynamical link between variations in deep convection or water mass formation and the AMOC are generally concerned with large scale, meridionally coherent AMOC variations. Often spatial filtering, such as empirical orthogonal functions, is applied to diagnose modes of AMOC variability [27,162,174,177,178], and the origin of these large-scale modes is studied. In other studies, an index of AMOC variability, such as the AMOC strength at $40^{\circ }\;\text{N}$, is used to quantify AMOC variations [25,177–179]. Such an index calculated in depth coordinates naturally focuses on modes of AMOC variability which have penetrated into the subtropics.

Observations, of course, are missing these large-scale spatial and temporal filters. In the subtropical gyre, wind-driven heaving of isopycnals and Ekman overturning cells project strongly onto the overturning (in both depth and density space). From a timeseries of the AMOC at a given latitude (e.g. $26^{\circ }\;\text{N}$), we do not know whether we are observing an anomaly in a large-scale overturning cell or a local wind-driven overturning cell. Modelling results [174] suggest that temporal filtering may remove much of the local wind-driven AMOC variability (figure 8*b*), which implies we may be able to isolate the large-scale AMOC variations if we observe long enough.

In the subpolar gyre, we are closer to the regions of water mass transformation, where buoyancy-driven AMOC variations are thought to originate. As a result, we might hope to better observe buoyancy-driven AMOC variations that reach the subtropics. Yet, most of the variations in overturning in the subpolar gyre never reach the subtropics. These AMOC variations are related to the water mass transformation occurring in the subpolar gyre circulations, and most of these waters are expected to be recirculated within the subpolar gyre. Even if we place floats within the DWBC in the UNADW layer, most of these floats are swept up in the subpolar gyre and are not exported to the subtropics [7,8,112]. As the case for the subtropical AMOC, we have no means of knowing whether an AMOC variation that we observe is restricted to the subpolar gyre or an AMOC anomaly that is likely to be exported to the subtropics.

Typically, AMOC variability is measured by variations in the strength of the lower limb ($\Psi_{\max}$). However, at subpolar latitudes, some of these lower limb waters will be contained within the mixed layer. Clearly, dense waters cannot enter the subtropics until these are subducted below the mixed layer. Therefore, we might instead focus on the volume transport of lower limb waters that are below the mixed layer; the volume transport of these waters might bear a stronger relationship with the waters that are exported to the subtropics.

It is possible that observations of the AMOC in the subtropical–subpolar transition zone could shed light on the origin of AMOC anomalies which enter the subtropics, including evaluating the meridional connection for the LNADW layer that is suggested to be more significant than in the UNADW layer [9,102,180]. Numerical and observational experiments are currently planned, including tall mooring and Lagrangian float deployments as well as high-resolution hydrography surveys, to specifically describe the processes that maintain or break the meridional propagation of signals through the transition zone between the Flemish Cap and the Tail of the Grand Banks.

## 5. Conclusion

In this article, we examine the structure of the mean AMOC and its variability in terms of the overturning in density space and the role of diapycnal water mass transformation. Because the isopycnals in the subpolar North Atlantic tilt strongly upward from east to west (western part of basin is colder than eastern part), and water masses tend to follow isopycnal rather than depth surfaces, the overturning in density coordinates gives a more accurate impression of the flow field than the overturning in depth coordinates (§2a and figure 1).

The overturning in density coordinates is related to diapycnal water mass transformation via the Walin framework [28]. The mean strength of the overturning in density space in the subpolar gyre is similar to that of surface water mass transformation, but the density of these waters is modified by diapycnal mixing. In particular, NADW is created by the mixing of OWs with lighter subpolar mode waters (§2b and figure 2).

Surface water mass transformation is best understood as a progressive densification as waters circulate cyclonically around the subpolar gyre (figure 3). The densest waters and the deepest convection occur at the two termini of this densification, the Labrador Sea and the Nordic Seas, but little water mass transformation occurs in the Labrador Sea, as waters are already extremely cold and freshening of waters compensates for cooling (§3a). The eddy-driven interaction between the convective interior and boundary currents is a key to water mass transformation and overturning in marginal seas (figure 4*a*). This interaction is important in explaining the mean residence timescale (several years for the Irminger and Labrador Seas) and the spread in residence timescales of dense waters in a marginal sea, as dense waters formed in or near the boundary current are exported rapidly, whereas dense waters that are formed in the interior must be transported by eddies into the boundary current prior to export (§3c).

To lead to large-scale AMOC variations, anomalies in NADW transport must be communicated to the subtropics (§3d). The majority of NADW formed in the subpolar gyre is not exported rapidly, but rather is recirculated within the energetic subpolar gyre circulation before being exported to the subtropics. Recirculation in the subpolar gyre is more prevalent for UNADW than for OWs, yet both UNADW and OWs reach the subtropics through both the Deep Western Boundary Current and complex interior pathways [102,112,114–181]. Recirculations within the subpolar gyre, interior pathways and mixing with older waters (1) lengthens the pathways to the subtropics and (2) results in a multitude of possible pathways. Thus, waters entering the subtropics are relatively old and have a large range of ages (figure 5).

While the mean overturning in density coordinates is strongly tied to the mean water mass transformation, the same is not true for interannual variations in the overturning. Changes in water mass transformation, volume changes between isopycnals and local export of dense waters are related via the Walin framework; the relationship between these terms depends on the timescale of variability, specifically whether it is shorter or longer than the residence timescale of NADW northward of the latitude of interest. If surface water mass transformation varies on timescales shorter than the residence timescale of NADW, it will result in changes in volume between isopycnals rather than export. As these volume changes can be later consumed locally, variability in the export of NADW (overturning in density space) is expected to be much less than the variability of the surface water mass transformation. For this reason, high-frequency atmospheric variations lead to changes in deep convection and the properties of NADW, but they are expected to have a much smaller impact on export. If water mass transformation varies slowly, on timescales longer than the residence timescale of NADW, these changes are expected to result in changes in export. Thus, we expect that persistent changes in water mass transformation due to, e.g. anthropogenic climate change, will result in changes in export.

There is little evidence that interannual variations in surface water mass transformation between OSNAP East and the Greenland–Scotland Ridge or between OSNAP West and Davis Strait results in variations in export (§4a and figure 7*a*,*b*), although relationships are likely stronger on longer timescales. Between $45^{\circ }$N and the Greenland–Scotland Ridge/Davis Strait interannual changes in surface water mass transformation are almost entirely compensated by volume changes between isopycnals, with export changes being much smaller (§4b and figure 7*c*). Given that in the mean, the total water mass transformation must be balanced by export, and one might expect that one could find a relationship between the variability of water mass transformation and export on some timescale longer than the residence timescale. However, the large range of residence timescales of NADW entering the subtopics (figure 5*c*) likely obscures finding a strong relationship, as waters entering the subtropics would be a mixture of waters formed at different times, during different conditions. Thus, only large and persistent (decadal and longer) changes in water mass transformation have the potential to be communicated to the subtropics [47,62,69,118,150].

The dilution of signals from upstream water mass transformation suggests that variability in export of dense waters to the subtropics may be controlled by other processes, in particular local or remote forcing in the western transition zone separating the subpolar and subtropical domains (§4c). There is some tantalizing evidence that interactions with the energetic upper ocean impacts NADW variability in this region. Ongoing observational campaigns may soon be able to address the variability in this region in more detail.

Models, particularly those with coarse resolution, may be missing the complex array of processes that lead to spreading of UNADW. As a result, downstream AMOC variability may be too tightly tied to upstream variations in source waters. In addition, models have a tendency to overproduce LSW and overestimate LSW variability (see §§2b and 4a), leading to an unrealistically strong relationship between LSW formation and export to the subtropics in models. Despite this, the nature of AMOC observations, which are limited to a few latitudes and a decade or two at most, will make it hard to observe relationships between variations in upstream water mass transformation and overturning, even if there are robust relationships between these quantities when large-scale spatial and long timescale temporal filters are applied, as is suggested by modelling results (§4b).

Understanding AMOC variability from limited observations is difficult. In the subtropical gyre large, local wind-driven AMOC variations obscure buoyancy-forced variations in the AMOC. In the subpolar gyre, one might hope to better observe buoyancy-driven AMOC variations, but much of the local overturning variability is related to the vigorous subpolar gyre circulation and not directly related to variations in export, at least on the timescales over which observations are available. Rather than focusing on the entire meridional transport of the lower limb, it might be possible to focus only on the transport of waters which reside below the mixed layer, which might bear a stronger relationship with the waters that are exported to the subtropics. It is possible that observations of the AMOC in the subtropical–subpolar gyre transition zone could better shed light on the origin of AMOC anomalies which enter the subtropics.

## Data Availability

The observation-based diagnosis of time-mean (2002–2017) water mass transformation and formation (figure 4) were obtained from [47]. They were built using the EN4 dataset (https://www.metoffice.gov.uk/hadobs/en4/ [57]) and an ensemble of atmospheric reanalysis products: NCEP2 (https://psl.noaa.gov/data/gridded/data.ncep.reanalysis2.html [59], ERA-I (now ERA5, www.ecmwf.int/en/forecasts/dataset/ecmwf-reanalysis-v5) and CERES (https://doi.org/10.17864/1947.111 [61]). ECCO v4 data used to produce figures 2, 3 and 8 was accessed via https://ecco-group.org/. Supplementary material is available online [182].
